# Supplementary material for: Oncogenic Role of ADAM32 in Hepatoblastoma: A Potential Molecular Target for Therapy
Source: Cancers (Basel). 2022 Sep 28;14(19):4732. doi: 10.3390/cancers14194732 (PMC9562177; doi:10.3390/cancers14194732)
Supplement: Supplementary file 1 [file cancers-14-04732-s001.zip › cancers-1932682-supplementary.pdf]

Supplementary Materials

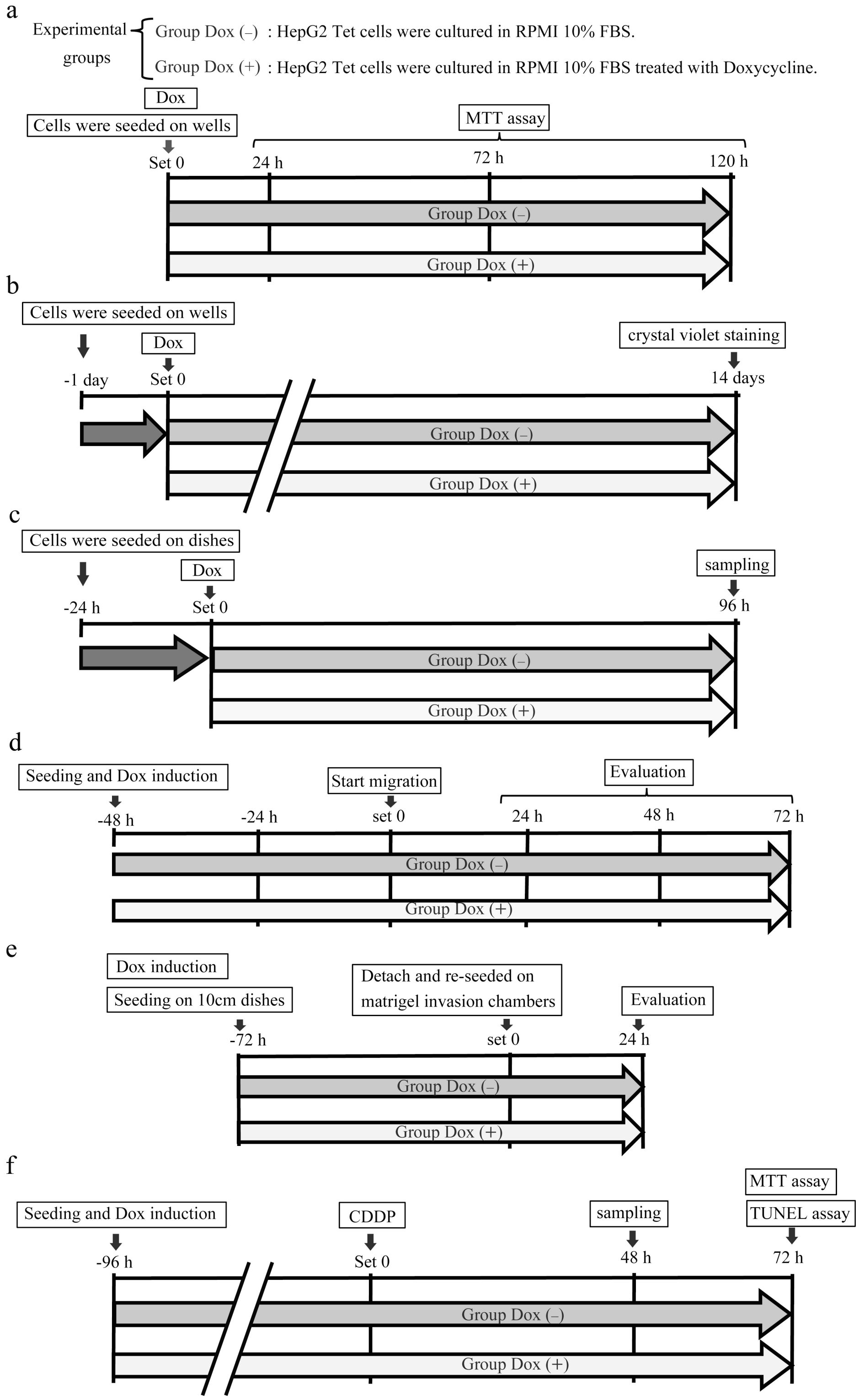

Figure S1. Detailed time schedules for ADAM32-forced expression experiments. (a) Sampling schedule for MTT assay, (b) colony formation assay, (c) real-time RT-PCR samples, (d) cell migration assay, and (e) cell invasion assay. (f) CDDP was added after ADAM32 expression was induced by Dox treatment. Sampling was then performed for immunoblotting and real-time RT-PCR at 48 h, and the MTT and TUNEL assays were conducted at 72 h.

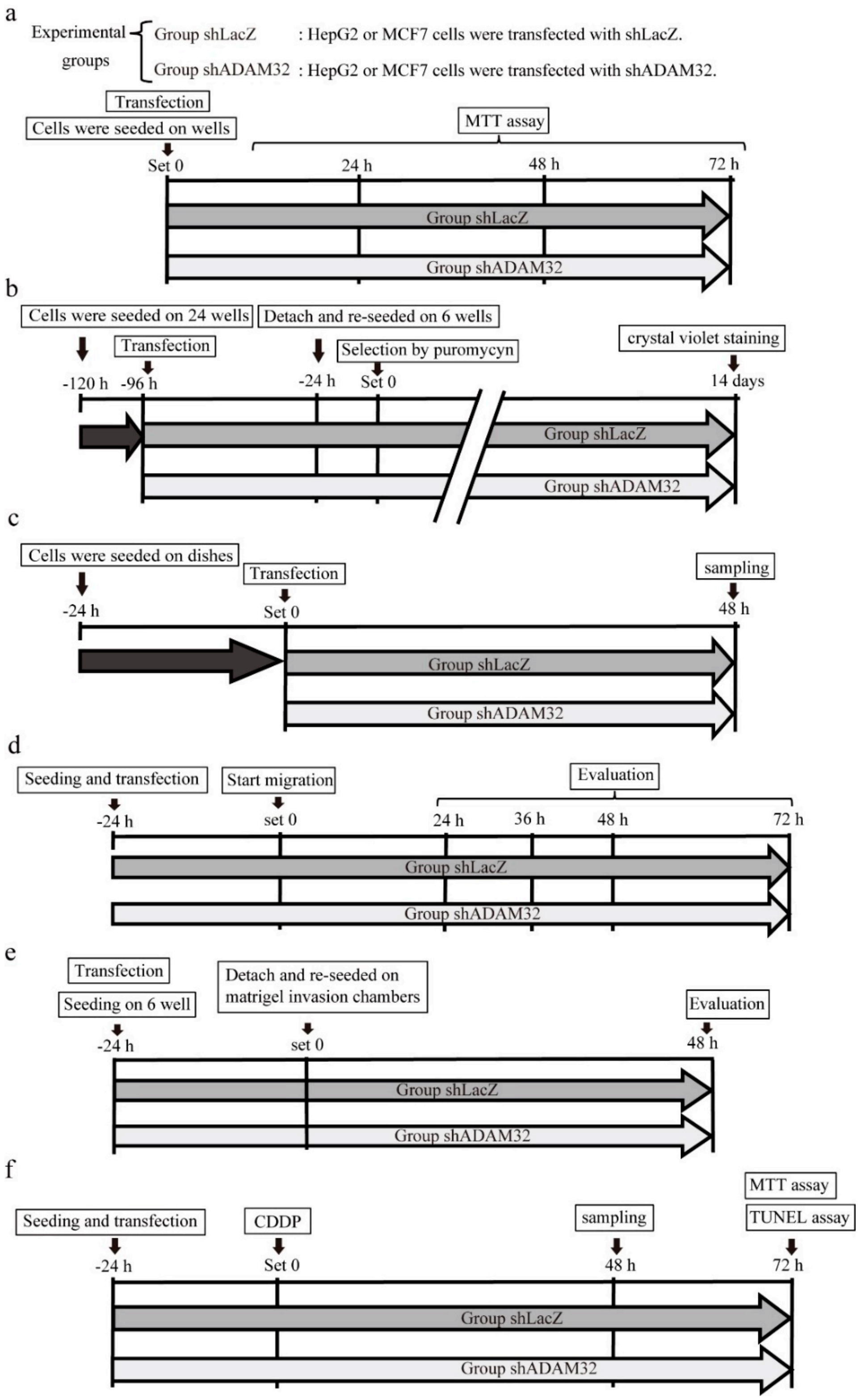

Figure S2. Detailed time schedules for ADAM32 knockdown experiments in HepG2 and MCF7. (a) Sampling schedule for MTT assay, (b) colony formation assay, (c) real-time RT-PCR samples, (d) cell migration assay, and (e) cell invasion assay. (f) CDDP was added after ADAM32 expression was silenced with shADAM32. Sampling was performed for immunoblotting and real-time RT-PCR at 48 h. MTT and TUNEL assays were conducted at 72 h.

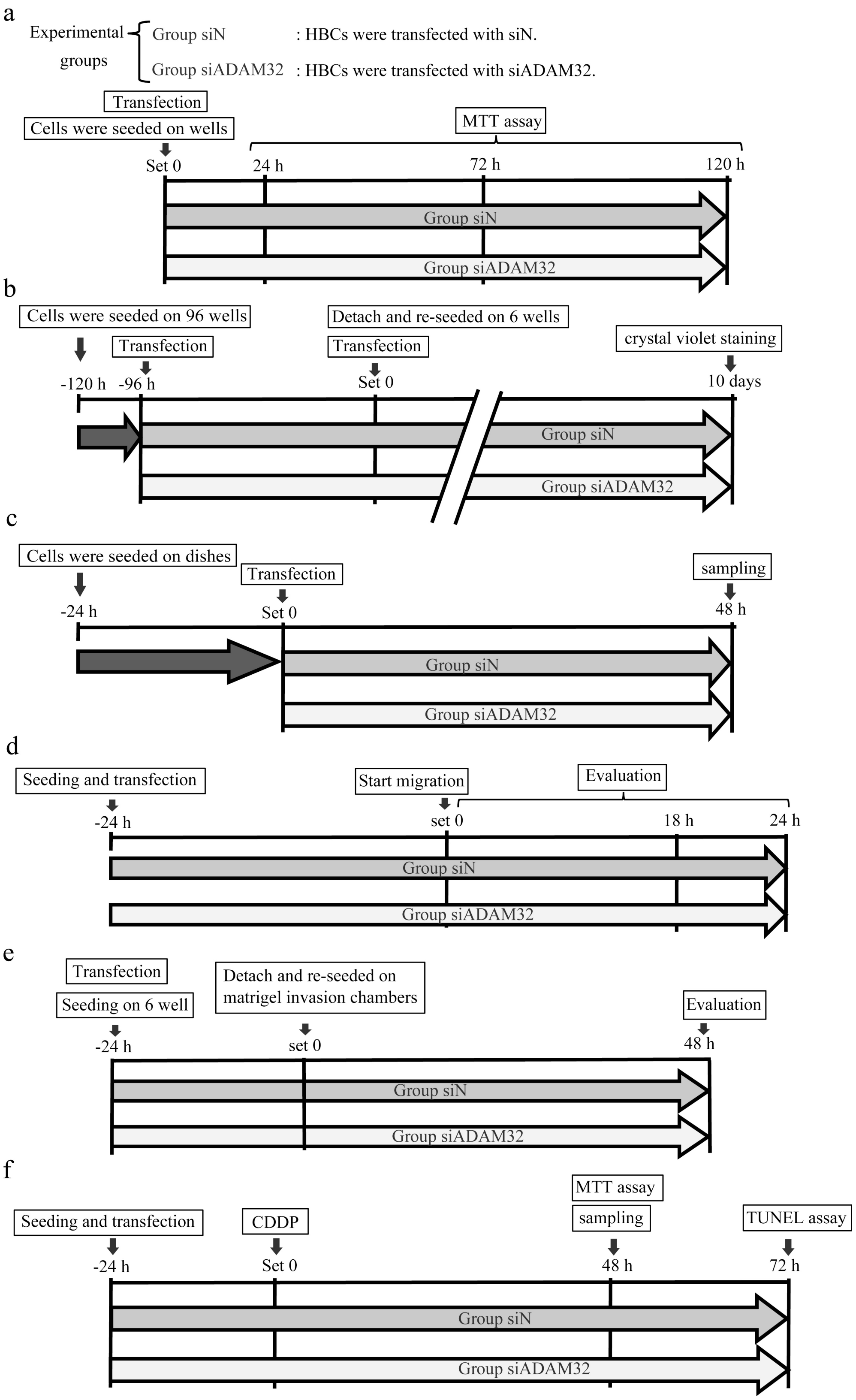

Figure S3. Detailed time schedules for ADAM32 knockdown experiments in HBCs. (a) Sampling schedule for MTT assay, (b) colony formation assay, (c) real-time RT-PCR samples, (d) cell migration assay, and (e) cell invasion assay. (f) CDDP was added after ADAM32 expression was silenced with siADAM32. MTT assay and sampling were performed for immunoblotting and real-time RT-PCR at 48 h. TUNEL assays were conducted at 72 h.

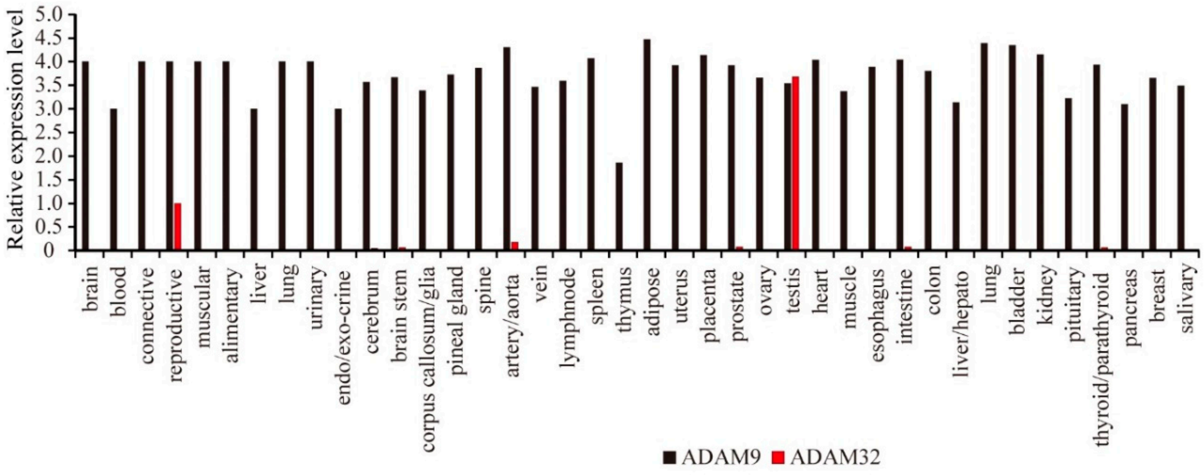

Figure S4. Specific expression of *ADAM32* in normal testis tissue. Relative expression levels (CAGE) of *ADAM9* and *ADAM32* in various normal tissues.

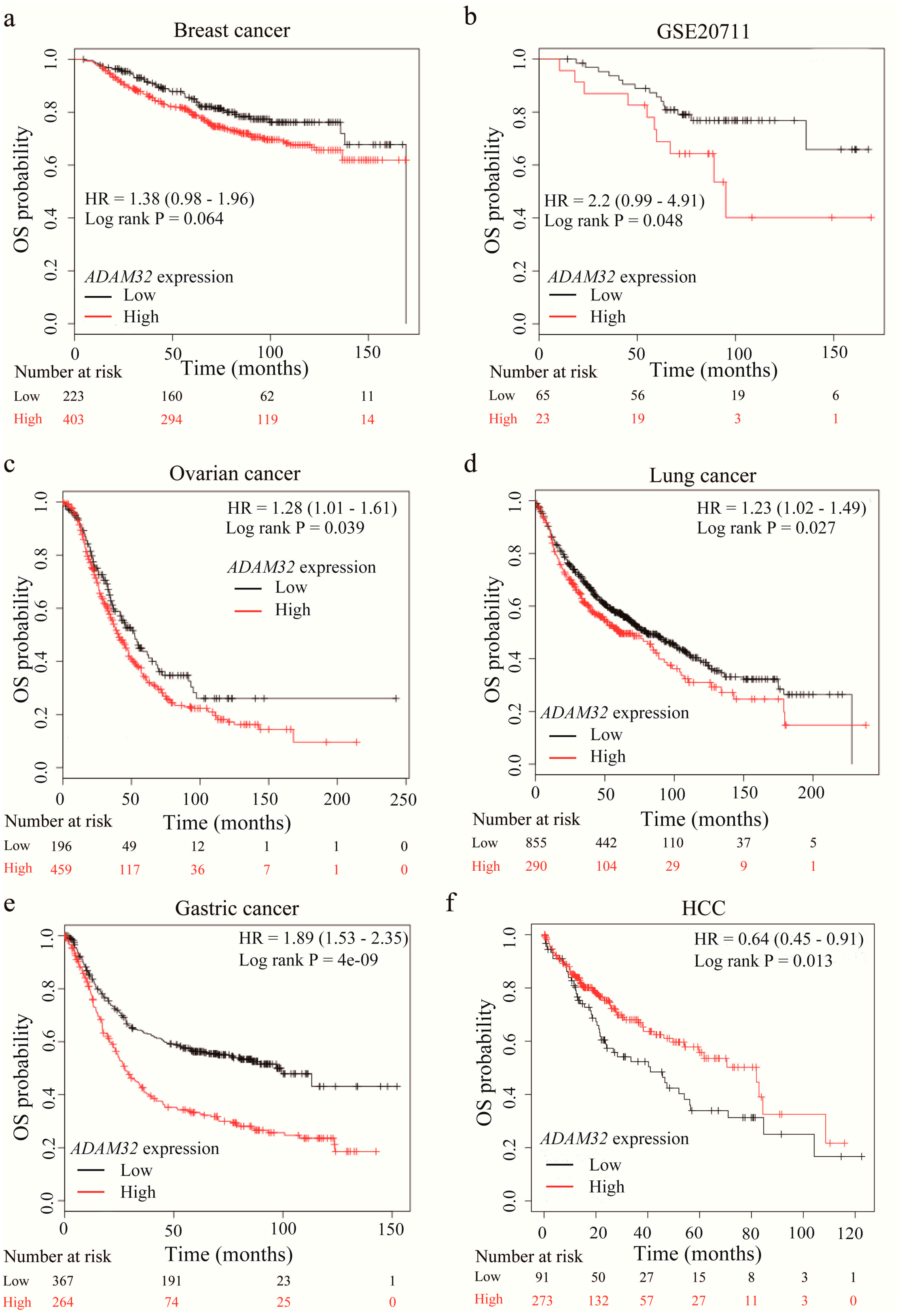

Figure S5. Prognostic association of ADAM32 in several cancer types. (a) The relationship between ADAM32 and OS was analyzed with the Kaplan-Meier plotter applied to gene chip data from patients with breast cancer. Gene chip data from patients with (b) breast cancer (GSE20711), (c) ovarian cancer, (d) lung cancer, and (e) gastric cancer. (f) RNA-seq data from patients with HCC. OS, overall survival; HR, hazard ratio

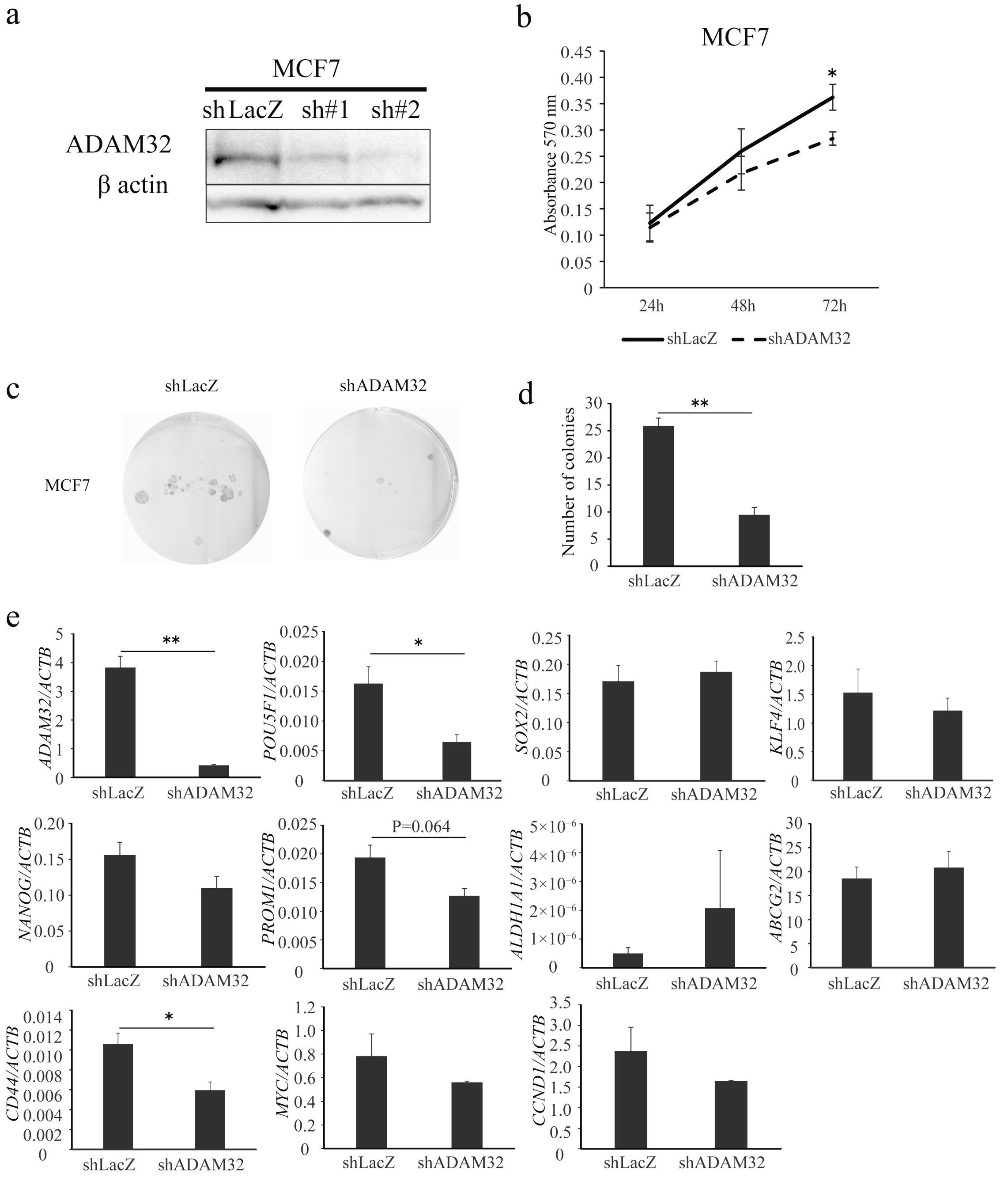

Figure S6. Roles of ADAM32 in cell viability and colony formation of breast cancer cells. (a) Immunoblotting showing expression levels of ADAM32 in MCF7 cells transiently transfected with shRNA. (b) Cell viability of MCF7 cells evaluated in the MTT assay (n = 3). (c) Representative images of MCF7 in the colony formation experiment. (d) The number of colonies was counted, and the average (and SE) are shown for MCF7 (n = 5). (e) The expression levels were evaluated with real-time RT-PCR by using total RNA prepared from MCF7 transiently transfected with shLacZ or shADAM32 and incubated for 48 h. Relative gene expression levels were calculated as the ratio relative to *ACTB* levels. Values are summarized by mean and SE (n = 3); *$p < 0.05$; **$p < 0.01$; Und, undetermined

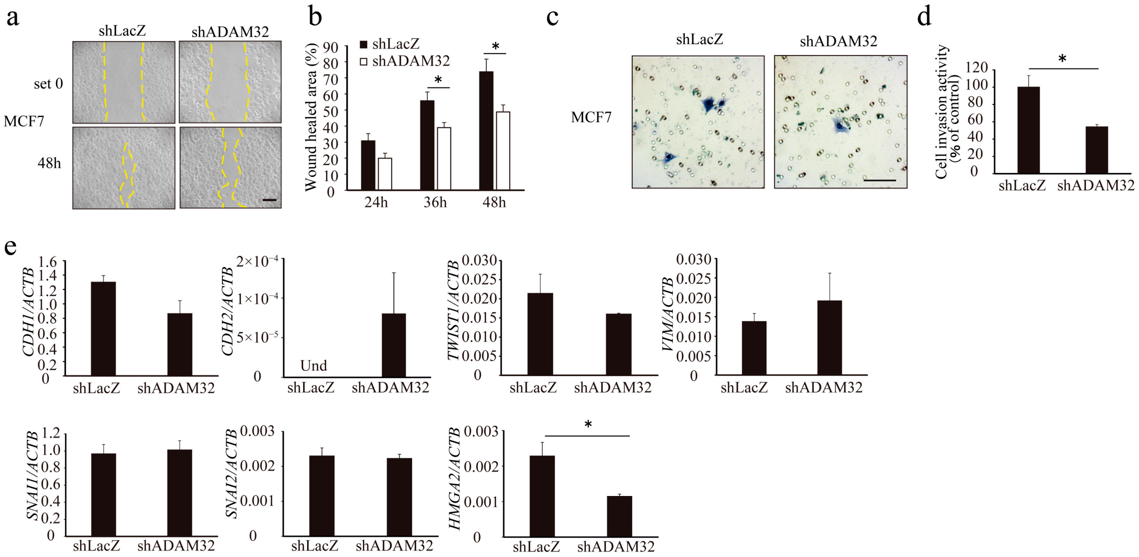

Figure S7. Roles of ADAM32 in cell migration and invasion in breast cancer cells. (a) Representative images of MCF7 transiently transfected with shLacZ or shADAM32 and incubated for 48 h in the wound-healing assays. (b) Calculated proportions of healed areas (%) are shown in bar graphs for MCF7 (n = 4). (c) Representative images of the cell invasion assay in MCF7 transiently transfected with shLacZ or shADAM32 and incubated for 48 h are shown. (d) The calculated ratios (%) of invading cell number to control in MCF7 are shown in bar graphs (n = 3). (e) Expression levels of EMT-related genes were evaluated with real-time RT-PCR by using total RNA prepared from MCF7 transiently transfected with shLacZ or shADAM32 and incubated for 48 h. Relative gene expression levels were calculated as the ratio to *ACTB* levels (n = 3). Scale bar = 200 μm. (b, d, e): Values are summarized by mean and SE. *$p < 0.05$; **$p < 0.01$; Und, undetermined.

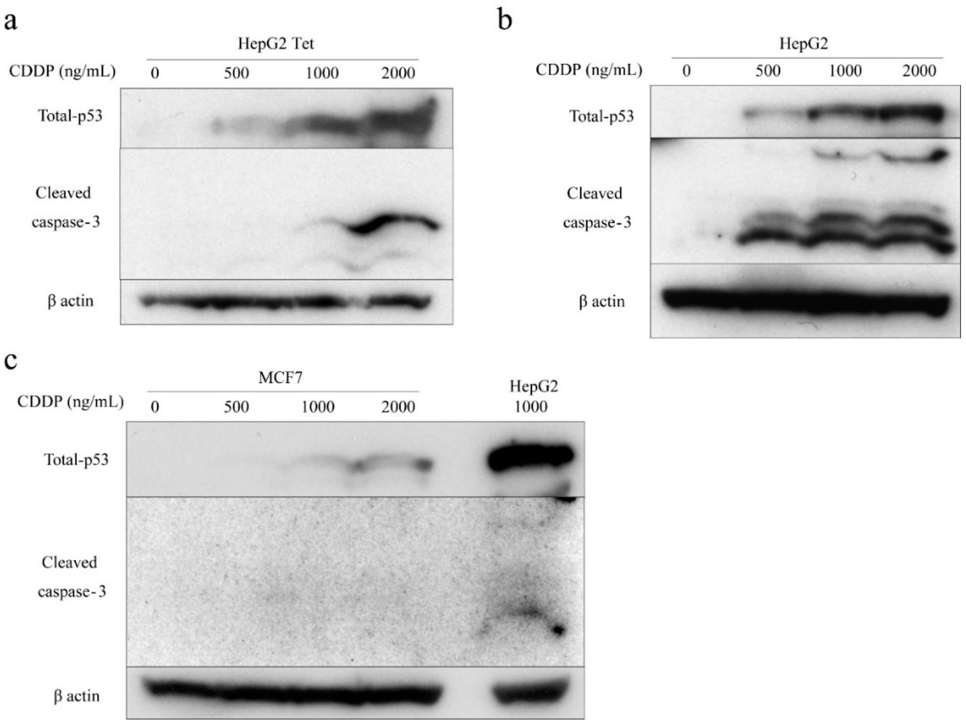

Figure S8. Concentration of CDDP used in this study was determined by immunoblotting. (a) HepG2 Tet cells treated with various concentrations of CDDP from 0 to 2000 ng/mL for 48 h. Levels of total p53 protein and cleaved caspase-3 were then evaluated by immunoblotting for (b) HepG2 and (c) MCF7. (a-c): Representative images from three independent experiments.

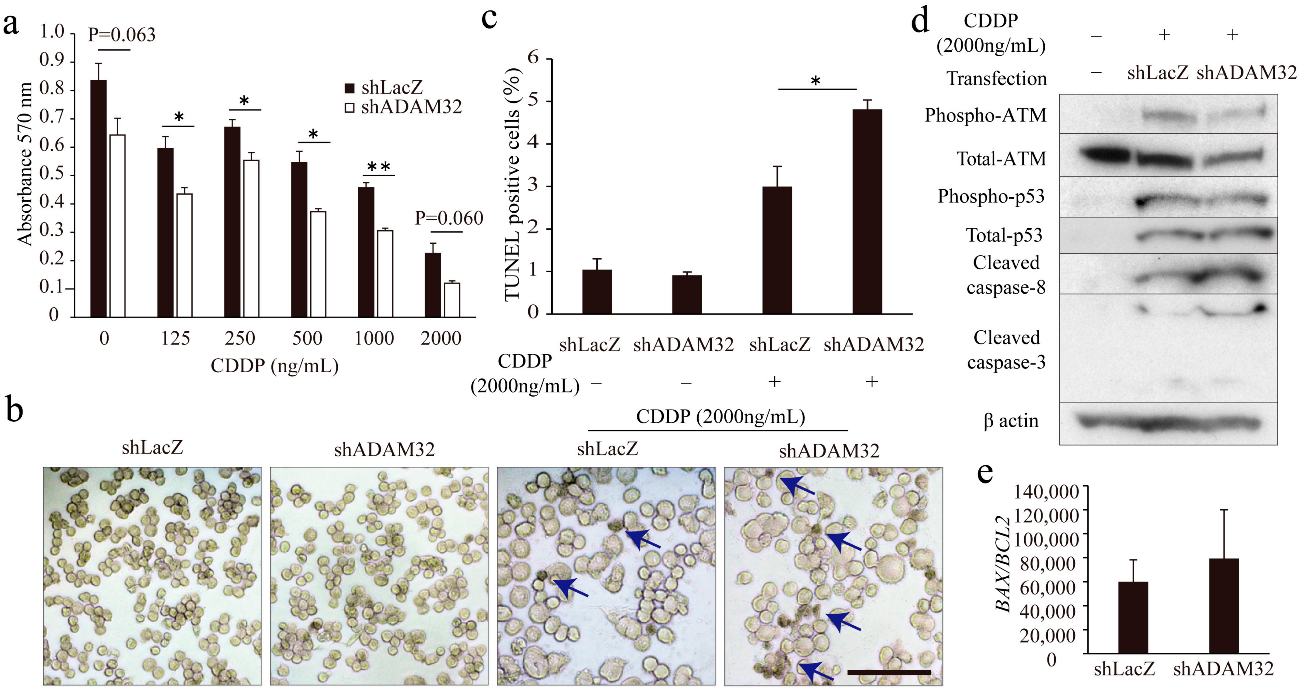

Figure S9. Anti-apoptotic functions of ADAM32 in CDDP-treated breast cancer cells. (a) MCF7 were transiently transfected with shLacZ or shADAM32 for 24 h and then treated with CDDP at various concentrations for 72 h. Cell viability in each group was evaluated with the MTT assay (n = 4). (b) Representative images are shown from the TUNEL assay using MCF7. (c) The percentages of TUNEL-positive cells are shown for MCF7 (n = 3). (d) MCF7 were transiently transfected with shLacZ or shADAM32 for 24 h and then treated with 2000 ng/mL of CDDP for 48 h. Expression levels of apoptosis-related proteins were evaluated by immunoblotting. (e) *BAX*/*BCL2* ratios are shown for MCF7 (n = 3). Values are summarized by mean and SE. *$p < 0.05$; **$p < 0.01$. (b): Blue arrows indicate TUNEL-positive cells. (d): Representative images are shown from three independent experiments. Scale bar = 100 μm.

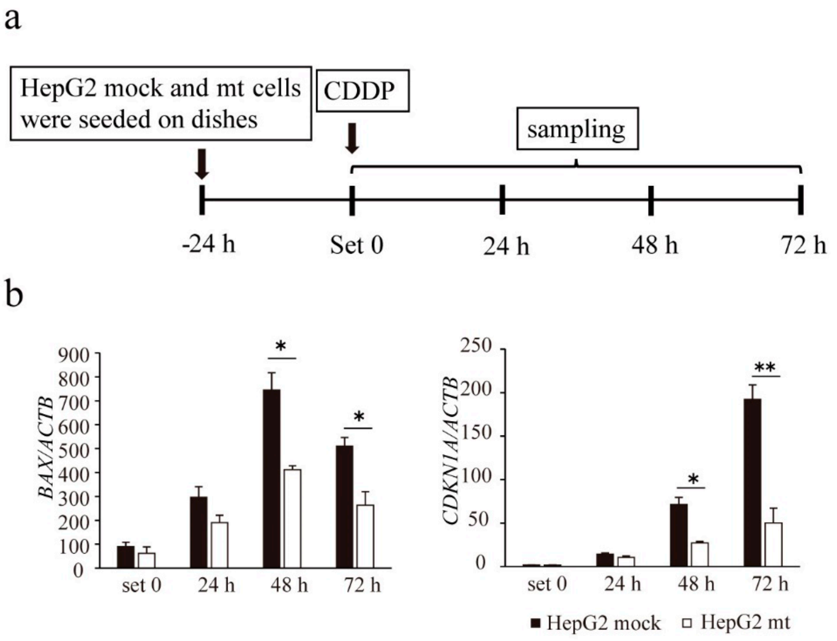

Figure S10. Inhibition of p53 signaling pathway in HepG2 mt cells. (a) HepG2 mock and HepG2 mt treated with CDDP at 500 ng/mL. Thereafter, expression levels of p53-targeted genes *BAX* and *CDKN1A* were evaluated by real-time RT-PCR by using the samples collected at 0, 24, 48, and 72 h. (b) Expression levels of *BAX* and *CDKN1A*. (b): Relative mRNA levels were calculated as ratio relative to *ACTB* levels. Values are summarized as mean and SE (n = 3); *$p < 0.05$; **$p < 0.01$

Table S1. Details of HBL cases and control tissues for IHC

Sample name	Tissue	Subtype	Sex	Age at diagnosis	PRETEXT	Metastasis
HBL1	HBL	Embryonal	F	1y 2m	III	+
HBL2	HBL	Embryonal	M	1y 11m	II	+
HBL3	HBL	Fetal	M	2y 3m	II	–
HBL4	HBL	Macrotrabecular	F	3y 7m	II	+
Normal liver	Normal liver	NA	M	13y	NA	NA

Table S2.

Oligo sets for plasmid construction

ADAM32	sense: 5′- ACCATGTTCCGCCTCTGGTTG -3′
	antisense: 5′- CTAGTTACTACTGCTTTGTGTTTGGG -3′

shRNA target sequences

Target sequence for shLacZ

5′- AGGTAAACAGTTGATTGAACTGC -3′

Target sequence for shADAM32 #1

5′- ATGAACAAATATCCTATATTATT -3′

Target sequence for shADAM32 #2

5′- CTGAAAGAAACAATAAATTGAGT -3′

Primer and probe sets for real-time RT-PCR

ADAM32	Forward: 5′- ACCTAAGGCCTCATGATATTGC -3′
	Reverse: 5′- TGCAAATGCCTCCAGAGTTA-3′
	Universal Probe Library: #32
POU5F1	Forward: 5′- CTTCGCAAGCCCTCATTTC-3′
	Reverse: 5′- GAGAAGGCGAAATCCGAAG-3′
	Universal Probe Library: #60
SOX2	Forward: 5′-TGCTGCCTCTTTAAGACTAGGAC -3′
	Reverse: 5′- CCTGGGGCTCAAACTTCTCT-3′
	Universal Probe Library: #35
KLF4	Forward: 5′- GGGAGAAGACACTGCGTCA-3′
	Reverse: 5′- GGAAGCACTGGGGGAAGT-3′
	Universal Probe Library: #52
NANOG	Forward: 5′-AGATGCCTCACACGGAGACT -3′
	Reverse: 5′- TTTGCGACACTCTTCTCTGC-3′
	Universal Probe Library: #31
PROM1	Forward: 5′- AACCTTACACGAGCAAGGAATTA-3′
	Reverse: 5′- AAACTTGTTCAAAAGTGAGCTTCAT-3′
	Universal Probe Library: #48
ALDH1A1	Forward: 5′- GCAACTGAGGAGGAGCTCTG-3′
	Reverse: 5′-GTCTTGCGGCCTTCACTG -3′
	Universal Probe Library: #88
ABCG2	Forward: 5′-TGGCTTAGACTCAAGCACAGC -3′
	Reverse: 5′-TCGTCCCTGCTTAGACATCC -3′
	Universal Probe Library: #56

CD44	Forward: 5′- CAACAACACAAATGGCTGGT-3′
	Reverse: 5′- CTGAGGTGTCTGTCTCTTTCATCT-3′
	Universal Probe Library: #40
MYC	Forward: 5′- CACCAGCAGCGACTCTGA-3′
	Reverse: 5′- GATCCAGACTCTGACCTTTTGC-3′
	Universal Probe Library: #34
CCND1	Forward: 5′- GCTGTGCATCTACACCGACA-3′
	Reverse: 5′-TTGAGCTTGTTCACCAGGAG-3′
	Universal Probe Library: #17
CDH1	Forward: 5′- GCCGAGAGCTACACGTTCA-3′
	Reverse: 5′- GACCGGTGCAATCTTCAAA-3′
	Universal Probe Library: #80
CDH2	Forward: 5′- CTCCATGTGCCGGATAGC-3′
	Reverse: 5′- CGATTTCACCAGAAGCCTCTAC-3′
	Universal Probe Library: #74
TWIST1	Forward: 5′- CCCAACTCCCAGACACCTC-3′
	Reverse: 5′- CAAAAAGAAAGCGCCCAAC-3′
	Universal Probe Library: #79
VIM	Forward: 5′- GTTTCCCCTAAACCGCTAGG-3′
	Reverse: 5′- AGCGAGAGTGGCAGAGGA-3′
	Universal Probe Library: #56
SNAI1	Forward: 5′- GCTGCAGGACTCTAATCCAGA-3′
	Reverse: 5′- ATCTCCGGAGGTGGGATG-3′
	Universal Probe Library: #11
SNAI2	Forward: 5′- TGGTTGCTTCAAGGACACAT-3′
	Reverse: 5′-GTTGCAGTGAGGGCAAGAA -3′
	Universal Probe Library: #7
HMGA2	Forward: 5′- TTCTCTCCTAGTGAGTGTGCTGAC -3′
	Reverse: 5′- TGACAAAAGCCGTCATGAGA-3′
	Universal Probe Library: #79
BAX	Forward: 5′- CCATCATGGGCTGGACAT-3′
	Reverse: 5′- CACTCCCGCCACAAAGAT-3′
	Universal Probe Library: #69
BCL2	Forward: 5′- TTGGTATCCTTCTCTTTCACGCAC-3′
	Reverse: 5′-ATGGCATTGACGAAGAGGAT -3′
	Universal Probe Library: #23
CDKN1A	Forward: 5′-TCACTGTCTTGTACCCTTGTGC -3′
	Reverse: 5′- GGCGTTTGGAGTGGTAGAAA-3′
	Universal Probe Library: #32

Table S3. Details of HBL case for primary cell culture

Subtype	Sex	Age at diagnosis	PRETEXT	Metastasis	Note
Mixed epithelial and mesenchymal	F	1y 4m	I	–	In addition to embryonal and fetal components, a mesenchymal component was also observed.

Table S4. Primary antibodies and dilutions

Antibody	Provider	Catalogue No	Dilution
anti-ADAM32	Sigma	HPA044156	1:500
anti-FLAG M2	Sigma	F3165	1:1000
anti-p53	ONCOGENE	OP43	1:1000
anti-phospho-p53	Cell Signaling Technology	#9284	1:500
anti-ATM	Cell Signaling Technology	#2873	1:1000
anti-phospho-ATM	Cell Signaling Technology	#5883	1:1000
anti-cleaved Caspase-3	Cell Signaling Technology	#9661	1:500
anti-Caspase-8	Cell Signaling Technology	#9746	1:500
anti-β-actin	Sigma	A5316	1:5000

Supplemental Material and Methods

Data mining using the Kaplan-Meier plotter

To confirm the relationship between ADAM32 expression and overall survival (OS), hazard ratio (HR) with confidence intervals and log rank *P* were calculated in Kaplan-Meier Plotter (http://kmplot.com/analysis) by using gene chip data from patients with breast, ovarian, lung, or gastric cancer, or RNA-seq data from patients with HCC. Details of each data sets are shown in the Kaplan-Meier Plotter.

Figure S11. Raw data of immunoblittings.

Figure 2a

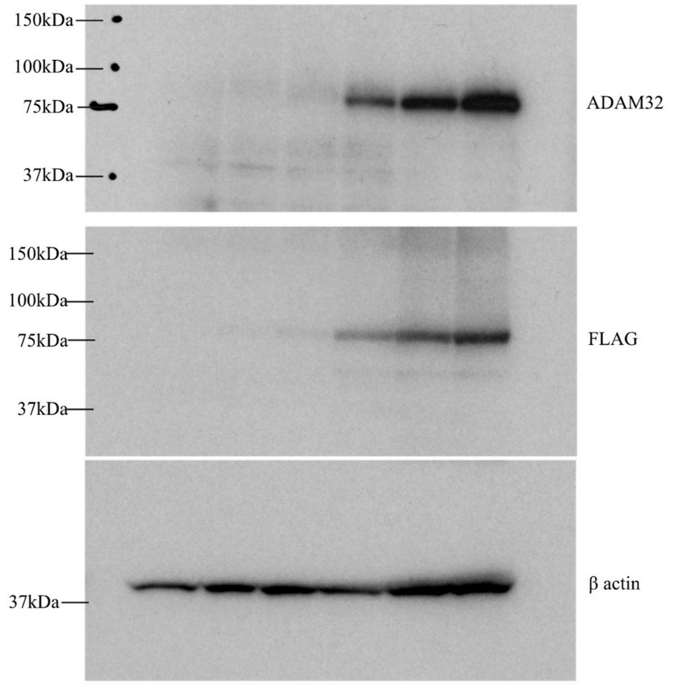

Figure 2b

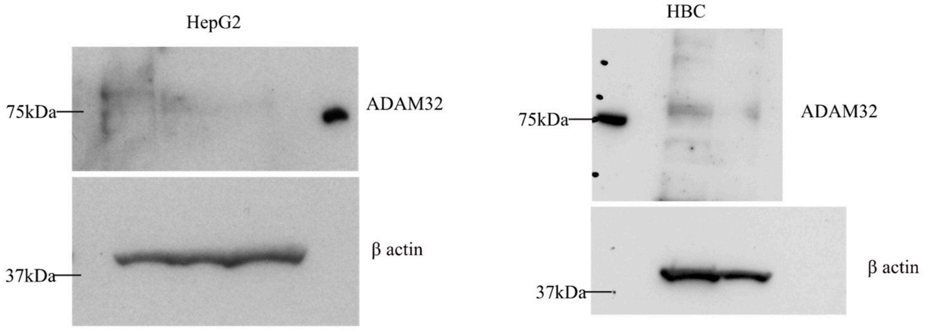

Raw data of immunoblot from Figure 2a and 2b.

Figure 4p

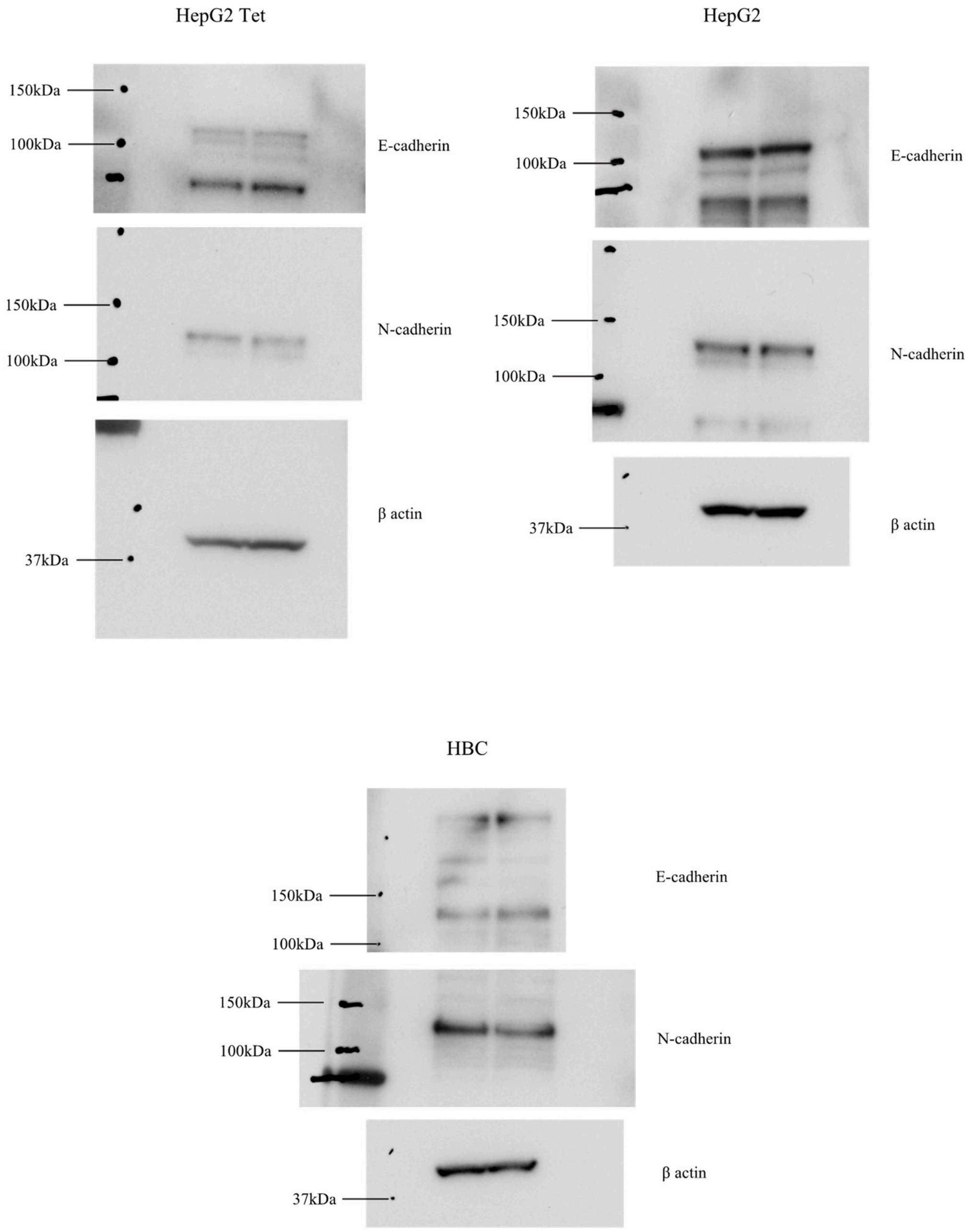

Raw data of immunoblot from Figure 4p.

Figure 5d

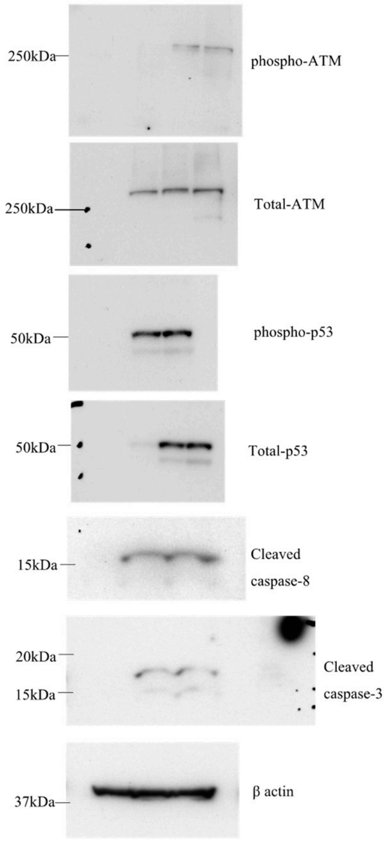

Figure 5i

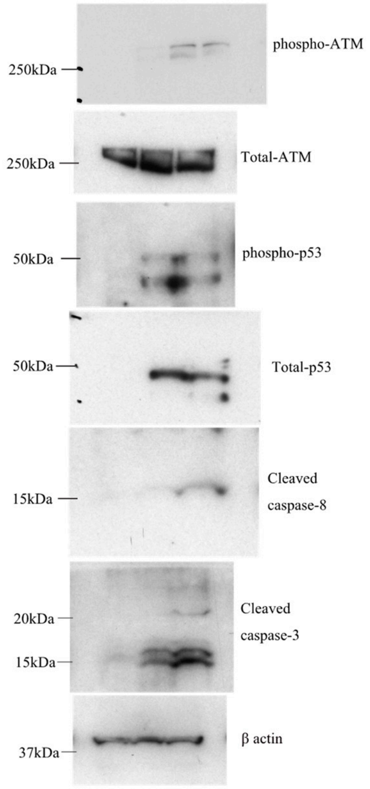

Figure 5n

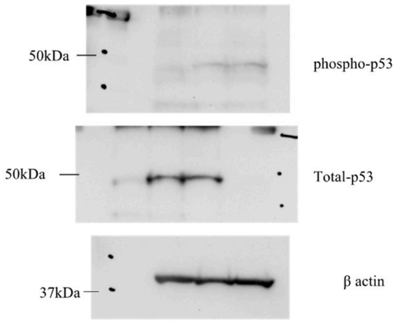

Raw data of immunoblot from Figure 5d, 5i and 5n.

Figure 6d

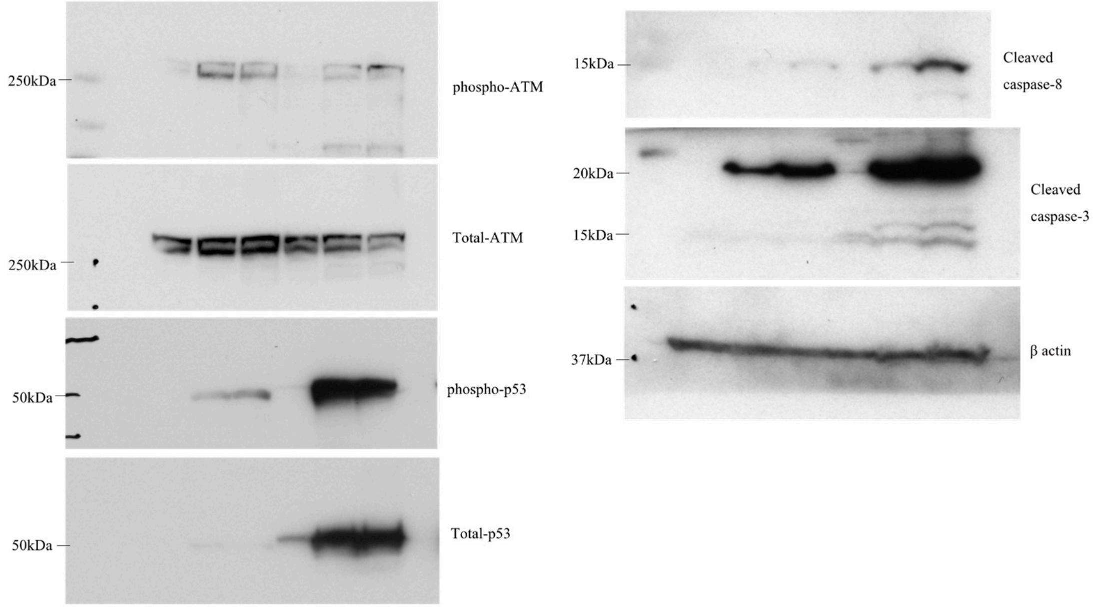

Figure 6i

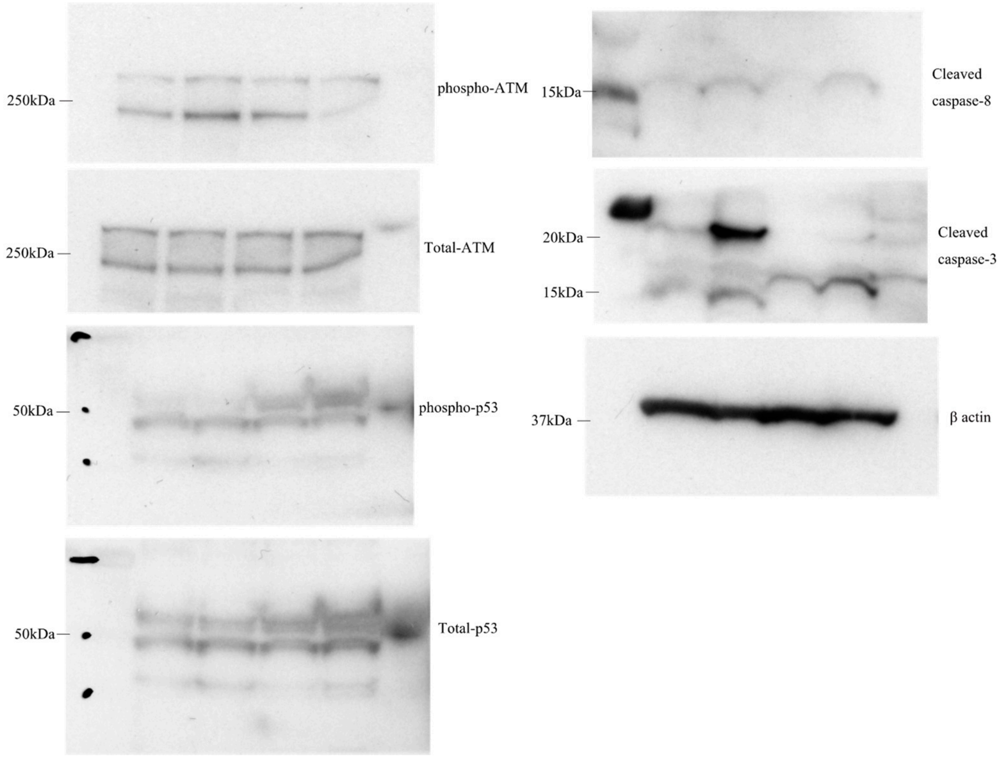

Raw data of immunoblot from Figure 6d and 6i.

Figure S6a

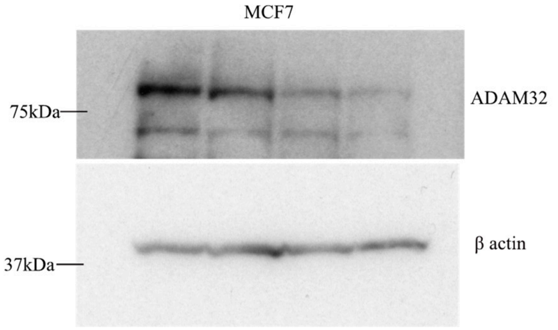

Figure S8a

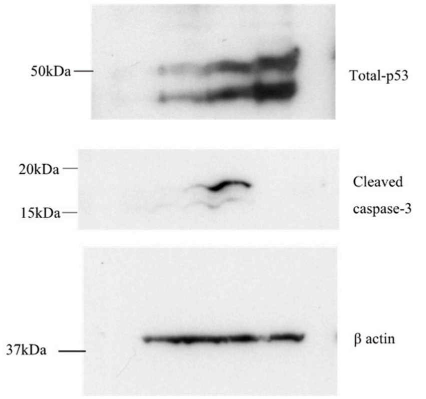

Figure S8b

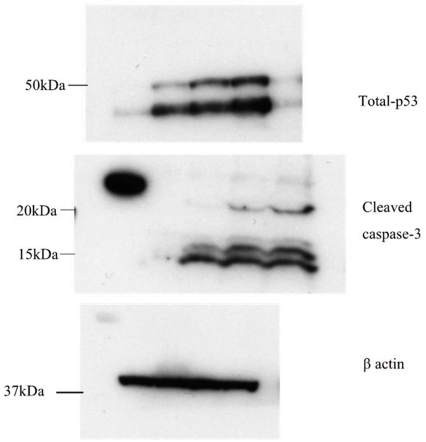

Figure S8c

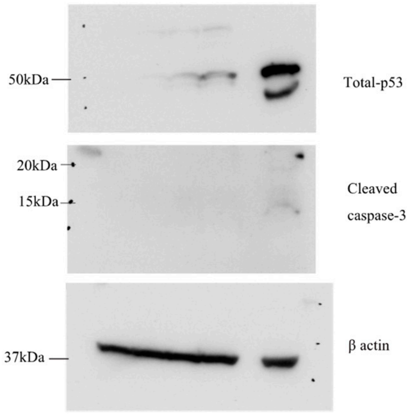

Raw data of immunoblot from Figure S6a, S8a, S8b and S8c.

Figure S9d

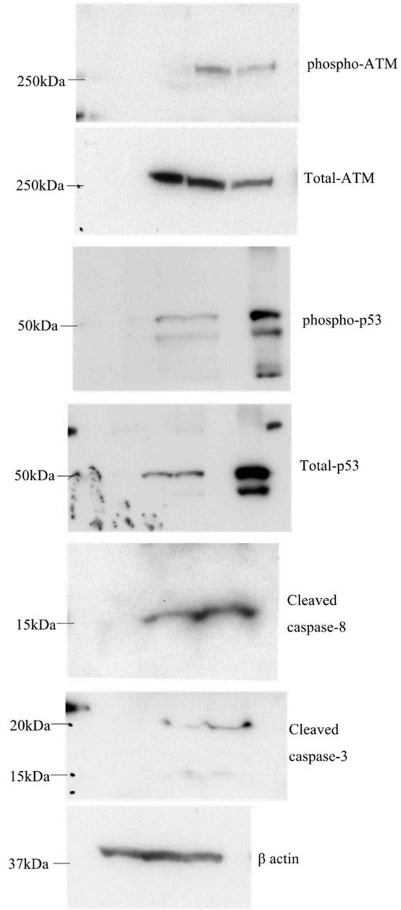

Raw data of immunoblot from Figure S9d.
